# Fast convolution-based performance estimation method for diffraction-limited source with imperfect X-ray optics

**DOI:** 10.1107/S1600577520012825

**Published:** 2020-10-23

**Authors:** Lingfei Hu, John P. Sutter, Hongchang Wang

**Affiliations:** a Diamond Light Source Ltd, Harwell Science and Innovation Campus, Didcot, Oxfordshire OX11 0DE, United Kingdom; b Beijing Synchrotron Radiation Facility, Institute of High Energy Physics, Chinese Academy of Sciences, 19B Yuquan Road, Shijingshan District, Beijing 100049, People’s Republic of China

**Keywords:** X-ray optics, wave optics, coherence, optical element error

## Abstract

Although optical element error analysis is always an important part of beamline design for highly coherent synchrotron radiation or free-electron laser sources, the usual wave optics simulation can be very time-consuming, which limits its application at the early stage of the beamline design. In this work, a new theoretical approach has been proposed for quick evaluations of the optical performance degradation due to optical element error. In this way, time-consuming detailed simulations can be applied only when truly necessary. This approach treats the imperfections as perturbations that convolve with the ideal performance. For simplicity, but not by necessity, the Gaussian Schell-model has been used to show the application of this theoretical approach. The influences of the finite aperture size and height error of a focusing mirror are analysed using the proposed theory. The physical explanation of the performance degradation acquired from the presented approach helps to give a better definition of the critical range of error spatial frequencies that most affect the performance of a mirror. An example comparing two mirror surface errors with identical power spectral density functions is given. These two types of mirror surface errors result in very different intensity profiles. The approach presented in this work could help beamline designers specify the error tolerances on general optical elements more accurately.

## Abstract 2

A theoretical approach is proposed to describe the performance degradation caused by imperfect X-ray optical elements.

## 1. Introduction   

Ever since the novel design of multiple-bend achromatic lattices (Einfeld *et al.*, 2014 ▸) made diffraction-limited synchrotron radiation sources possible, many synchrotron radiation sources are being upgraded in order to provide more coherent X-ray beams (Chenevier & Joly, 2018 ▸; Leemann *et al.*, 2018 ▸; Pellegrini, 2016 ▸; Shi *et al.*, 2017 ▸). In parallel, X-ray free-electron lasers have come to provide excellent spatial and temporal coherence in the X-ray region as well as at visible wavelengths. However, the great advances in coherent X-ray sources make the tolerances on optical elements more stringent if the high coherence originating at the source is to be preserved throughout the beamline.

An important aspect of beamline design is to study the effect of optical element errors. At beamlines of third-generation synchrotrons, the impact of imperfect optical elements can be evaluated by geometrical optics using ray-tracing simulation software (Baumgärtel *et al.*, 2016 ▸; Bergbäck Knudsen *et al.*, 2013 ▸; Klementiev & Chernikov, 2014 ▸; Rebuffi & Sanchez del Rio, 2016 ▸, 2017 ▸). However, for diffraction-limited synchrotron radiation (DLSR) or X-ray free-electron lasers (FEL), because of the low emittance of the source, the wave optics need to be taken into consideration. Wave optics simulation codes calculate wavefront propagation using the Fresnel diffraction integral. Several types of methods for wavefront propagation calculation have been used by different simulation codes. Among them are the wave-optics-based code *SRW* (Chubar & Elleaume, 1998 ▸) and *WISE* (Raimondi & Spiga, 2015 ▸), hybrid method based codes *xrt* (Klementiev & Chernikov, 2014 ▸) and others which have already been integrated into the widget-based interface *OASYS* (Rebuffi & Sanchez del Rio, 2017 ▸; Shi *et al.*, 2014 ▸). Although wave optics simulations yield more accurate results than ray tracing for DLSR and X-ray FELs, they usually require substantially more time unless carefully optimized. Some improvements have been made to make wave optics simulations faster (Sanchez del Rio *et al.*, 2019 ▸), but they still demand considerable computing resources. This drawback limits the use of wave optics simulations. As a result, a theoretical tool to evaluate the performance of imperfect optical elements prior to a detailed wave optics simulation will be useful.

Instead of numerical simulation, some theoretical discussions on the beamline performance have also been given by many researchers. Some discussed the coherence properties influenced by finite aperture size (Shi *et al.*, 2017 ▸; Singer & Vartanyants, 2014 ▸). Many others (Church & Takacs, 1993 ▸; Harvey, 1995 ▸; Harvey *et al.*, 1995 ▸; Raimondi & Spiga, 2015 ▸; Spiga, 2018 ▸) have discussed mirror surface specifications for third-generation synchrotron radiation as well. Two statistical parameters have been proposed to describe the mirror performance degradation, namely the root mean square (RMS) mirror height error and RMS residual slope error. However, wave optics simulations (Pardini *et al.*, 2015 ▸; Shi *et al.*, 2016 ▸) have already shown that these statistical parameters are not appropriate for the specification of DLSR or FEL beamlines. In particular, some side peaks often appear when the optical performance is simulated using the highly coherent source. It is very difficult to explain the appearance of these side peaks if the mirror’s imperfections are described only by RMS height and slope errors. Thomasset & Polack (2008 ▸) and Yashchuk *et al.* (2015 ▸) had already pointed out that mirror imperfections within a certain spatial frequency range cause more severe distortion of the focal spot and more intense side peaks than do mirror imperfections with spatial frequencies above or below this range. However, the definition of low frequency range in these early works is ambiguous. Raimondi & Spiga (2015 ▸) have done similar work. They investigated the performance degradation from imperfect mirrors in detail through both analytical expression and numerical simulation in terms of the point spread function (PSF) of the mirror.

In this work, a theoretical approach to evaluate the optical performance degradation caused by imperfect optical elements without using wave optics simulations is given. The proposed theory could be used to evaluate the impacts of finite size aperture, surface height error and other imperfections of optical elements as long as they can be described by a complex transfer function. Furthermore, the presented theory provides physical insights that help to explain the degradation of optical performance. These physical explanations will help beamline designers estimate the tolerances on their optical elements more accurately.

We will begin with a very concise introduction of the optical coherence theory. After that, the main theoretical results that are to be used throughout this paper will be given. Then two cases, finite aperture size and mirror surface height error, will be chosen to apply the proposed theory. Apart from the calculation of the cross spectral density function, we also provide physical explanations of the intensity profile distortion due to the mirror surface height error modulation. A summary of the proposed theoretical approach will be given at the end.

## 2. Perturbation theory for partially coherent beams   

### 2.1. Basic treatment of coherence   

The coherence of the light beam could be described in phase space by the Wigner distribution (Bazarov, 1987 ▸; Tanaka, 2017 ▸) or in spatial coordinate space (Schroer & Falkenberg, 2014 ▸; Singer & Vartanyants, 2014 ▸; Vartanyants & Singer, 2010 ▸). In this work we choose the spatial coordinate description. The treatment of coherence could be described well by the mutual coherence function and other related functions derived from it (Born & Wolf, 2013 ▸; Mandel & Wolf, 1995 ▸). The mutual coherence function is defined as
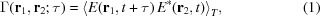
where 〈…〉*_T_* means an averaging over a long period of time *T*, *E*(**r**_1_, *t* + τ) and *E*(**r**_2_, *t*) are the complex amplitudes of wavefields at different positions **r**_1_, **r**_2_ and time *t* + τ, *t*. The mutual coherence function represents the correlation of the wavefield at two different positions and times. In this article, we restrict ourselves to the discussion of spatial (transverse) coherence rather than temporal (longitudinal) coherence. Furthermore, the electromagnetic field is assumed stationary. The assumption of stationary or quasi-stationary field is valid in most synchrotron radiation sources of hard X-rays (Geloni *et al.*, 2008 ▸, 2015 ▸; Kim, 1989 ▸). To discuss the spatial coherence of the synchrotron radiation, it is convenient to introduce the cross-spectral density (CSD) function, which is defined as the Fourier transform of the mutual coherence function
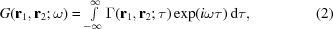
where ω is the frequency of the radiation. The normalized cross-spectral density function is called the spectral degree of coherence (SDC), denoted as μ(**r**_1_, **r**_2_; ω),
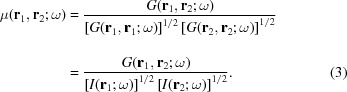
As in previous works (Geloni *et al.*, 2008 ▸; Kim, 1989 ▸; Schroer & Falkenberg, 2014 ▸; Singer & Vartanyants, 2014 ▸; Vartanyants & Singer, 2010 ▸), from now on, we will discuss the spectral functions, omitting the ω for simplicity.

The free space propagation of the cross-spectral density function from the initial plane *G*_0_(**r**_1_, **r**_2_; *z* = 0) at *z* = 0 to the downstream plane *G_z_*(**r**_1_, **r**_2_; *z* = *z*_0_) at *z* = *z*_0_ is well known to obey the following relation (Born & Wolf, 2013 ▸; Mandel & Wolf, 1995 ▸),
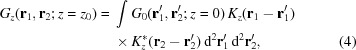
where *K*_*z*_(**r**) is the Fresnel propagator along the optical axis. Under the assumption of the paraxial approximation, *K*_*z*_(**r**) has the expression
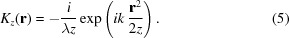



### 2.2. Propagation through a non-ideal optical surface   

**Figure 1:**
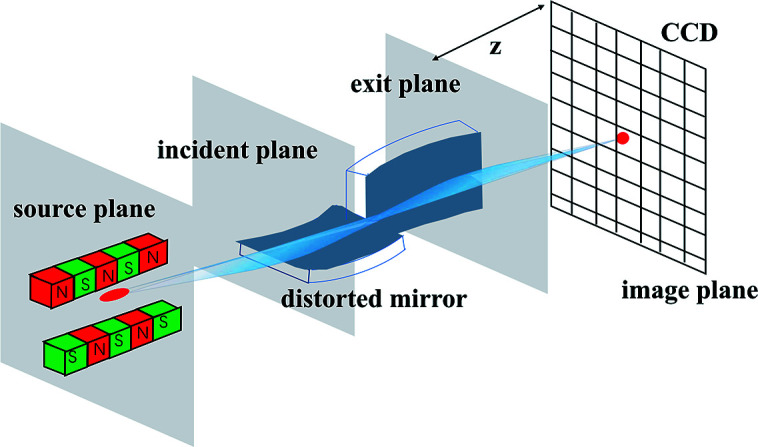
Schematic of the geometric layout for reflecting mirror.

The propagation of the CSD function through free space is governed by equation (4). For simplicity and without loss of generality, we only consider one transverse direction in equation (4) hereafter. An ideal optical surface may be defined as a surface of infinite extent with the ideal physical shape for beam profile shaping. To be more specific, an ideal infinite plane mirror only deflects the incident beam such that the reflected beam propagates as a free space diffraction along its reflected direction. An ideal infinite focusing mirror images the source according to its demagnification factor. On the other hand, a non-ideal optical surface deviates from the ideal shape because of height error and finite physical size. As shown in Fig. 1 ▸, the CSD function on the image plane should be derived from the CSD function on the exit plane close to the mirror through free space propagation according to equation (4). For an ideal optical surface, we have the one-dimensional version of equation (4),
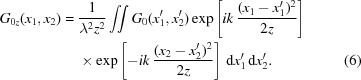

*G*_0*z*_(*x*_1_, *x*_2_) is the CSD function on the image plane through the ideal optical surface, *G*_0_(*x*_1_, *x*_2_) is the CSD function on the exit plane right after the ideal optical surface, *z* is the distance between the image plane and the exit plane, λ is the wavelength of the beam, and *k* = 2π/λ is the wavevector. The coordinates 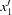 and 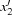 in the above integral lie within the exit plane.

In general, the non-ideal optical surface multiplies the ideal amplitude by a complex transfer function *t*(*x*′). The effects represented by *t*(*x*′) may include partial transmission due to mirror reflectivity, finite mirror size, mirror surface height error, *etc*. The specific expression of *t*(*x*′) related to these factors will be discussed later. By multiplying the complex amplitude of the incident beam by the complex transfer function *t*(*x*′), one obtains an equation similar to (6),
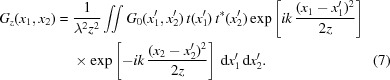
The basic idea of our new treatment is to separate equation (7) into two terms, one corresponding to the ideal optical element and the other related to the imperfection. The non-ideal optical element is hence a perturbation of the ideal optical element. A similar idea can be found in other articles (Tayabaly *et al.*, 2016 ▸; Raimondi & Spiga, 2015 ▸) where the intensity perturbation is considered. Here, the more general CSD function perturbation is given. After some mathematical derivation from equation (7) and comparing the result with equation (6), we state that the CSD functions at the image plane through the non-ideal optical surface and through the ideal optical surface are related as follows,
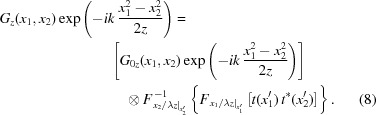
Appendix *A* [App appa] gives a more detailed mathematical derivation of equation (8). In the above equation, *G*_0*z*_(*x*_1_, *x*_2_) is the ideal CSD function at the image plane calculated from equation (6) and *G*_*z*_(*x*_1_, *x*_2_) is the non-ideal CSD function at the image plane calculated from equation (7), respectively. *F_υ_* and 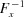 denote the Fourier and inverse Fourier transform. Specifically, 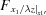 denotes a Fourier transform from 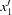 into *x*_1_/λ*z*. Similarly, 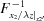 denotes an inverse Fourier transform from 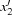 into *x*_2_/λ*z*. The symbol ⊗ denotes the 2D convolution. The 2D convolution, Fourier and inverse Fourier transforms are defined below:
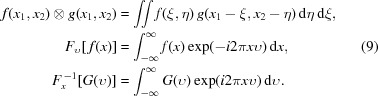
If *x*_1_ *= x*_2_, the CSD function *G*_*z*_(*x*_1_ *, x*_2_) becomes the intensity *I*(*x*_1_) at coordinate *x*_1_ of the image plane. It is sometimes convenient to change the variables of *x*_1_ and *x*_2_ to *x*_1_/λ*z* and *x*_2_/λ*z*. Therefore, the intensity at image plane can be written as the function of new variables,
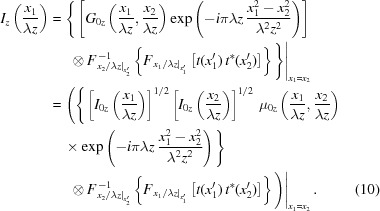

*G*_0*z*_(*x*_1_/λ*z*, *x*_2_/λ*z*) above is expanded according to equation (3). *I*_0*z*_(*x*_1_/λ*z*) and *I*_0*z*_(*x*_2_/λ*z*) are the ideal intensities at, respectively, positions *x*_1_ and *x*_2_ on the image plane. μ_0*z*_(*x*_1_/λ*z*, *x*_2_/λ*z*) is the SDC at the image plane. The *x*_1_ = *x*_2_ outside the curly bracket means setting *x*_1_ = *x*_2_ after the operation inside the bracket.

Equations (8) and (10) give us a general analytical description of the imperfections’ impacts on the CSD function and the intensity distribution. The 2D version of these results can be found in Hu *et al.* (2020 ▸). For the most general form of equations (8) and (10), 4D integration (convolution) is unavoidable. In that case, a special procedure such as coherent mode decomposition (Glass & Sanchez del Rio, 2017 ▸; Sanchez del Rio *et al.*, 2019 ▸) must be applied to reduce the computational burden. However, although the computationally demanding wave optics simulation in 2D is theoretically the correct procedure, the semi-analytical discussion and reduced 1D calculation are also important and helpful to investigate the impacts of imperfect optical elements. Thanks to the extensive use of Fourier and inverse-Fourier transforms and 2D convolution in computer science, there exist well established methods for calculating them rapidly. As a result, the fast Fourier transform (FFT) and FFT-based 2D convolution can be used for the calculation of equations (8) and (10). In particular, if the horizontal and vertical properties of the source are decoupled, as in the Gaussian Schell-model, the time needed to calculate equations (8) and (10) is negligible (Hu *et al.*, 2020 ▸). If only a 1D calculation is required, as is the case for grazing-incidence mirrors, equations (8) and (10) can be applied easily as well.

Apart from providing a new simulation method, equations (8) and (10) also provide physical insights into the performance degradation caused by the imperfections of optical elements. Later in this paper, these two expressions will help to show the underlying physical mechanism of the performance deterioration of imperfect mirrors. Moreover, although accurate simulation using wave optics is essential, a quick and reliable method for estimating the performance degradation could be used to screen the metrology data on a large number of optics to find those worthy of consideration for a high-quality DLSR. These two equations are general as long as the imperfections can be described by a complex transfer function. In the following sections, several specific examples of common imperfections on optical elements will be discussed.

## 3. Partially coherent X-ray beam after imperfect focusing optical element   

### 3.1. Focusing with perfect optical elements   

In order to use equations (8) and (10) to explore the impact that comes from optical imperfections, we need the ideal performance of the perfect optical element. We state here that the perfect optical element images the source according to the magnification factor *M*. No loss of intensity and no distortion of the intensity profile will occur at the final image plane. For the convenience of the discussion throughout this paper, we use the Gaussian Schell-model (GSM) to describe the DLSR or FEL source (Schroer & Falkenberg, 2014 ▸; Vartanyants & Singer, 2010 ▸). The advantage of the GSM is that the CSD function of the source can be written analytically given just a few parameters describing the properties of the source. We stress that the GSM is used here only for simplicity and because it is especially well suited for synchrotron and FEL sources. Any other model for the CSD could be used without any change to the procedure of this paper.

The CSD function of a GSM source is
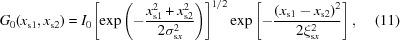
where *I*_0_ represents the maximum intensity at the source, and σ_s_ and ξ_s_ represent the source’s RMS size and coherence length, respectively. *x*_s1_ and *x*_s2_ are the coordinates at the source plane. Equation (11) shows that to describe the DLSR or FEL source using the GSM, two parameters, *i.e.* source size and coherence length, are required. Within the framework of the GSM, these two parameters have the following relation,
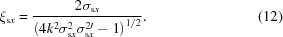
Here 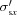 is the angular divergence of the source. Usually, the product 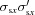 is defined as the beam emittance. The CSD function of the wave transmitted by a perfect focusing element is affected only by the magnification factor as follows. We have the following simple relations,
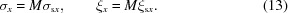
The CSD function at the image plane of the perfect optical element could be written as
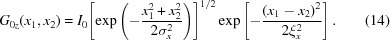
Here *x*_1_ and *x*_2_ are the coordinates at the image plane. The image size and image coherence length are derived from the same parameters from the source according to equation (13). The perfect CSD function at the image plane, which is described in equation (14), will be used throughout this paper. According to the outline in Section 2, the perfect CSD function will be ‘perturbed’ by the imperfection of the optical element. The imperfections from optical element include the finite aperture, the surface height error, *etc*.

The source parameters to be used in this paper are from the High Energy Photon Source (HEPS) project (Jiao *et al.*, 2018 ▸). We choose the horizontal direction for our discussion. Two typical focusing modes are considered. The first is the diffraction-limited focusing mode. In this mode, the source-to-mirror distance is 130.25 m and the image-to-mirror distance is 0.11 m. This makes the demagnification factor about 1184. The focal spot size is of the order of tens of nanometres. The second is the 1:1 focusing mode. The mirror is placed at 38.5 m from the source and the image plane is also located at 38.5 m from the mirror. If the optical element is perfect in this mode, what we obtain in the focal plane is exactly 1:1 with the source. Table 1 ▸ gives the detailed parameters used in this paper.

### 3.2. Optical elements with finite aperture   

A perfect optical element has an infinitely large aperture size. However, a real optical surface always has a finite physical size. The complex transfer function *t*(*x*′) to describe the finite optical element aperture at the exit plane could be expressed as a rectangular function,
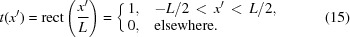

*L* is the size of the optic aperture projected onto the exit plane. According to equation (8), the CSD function after the finite aperture *G_z_*(*x*_1_, *x*_2_) has the following equation,
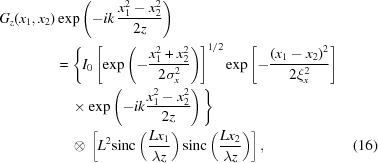
where sinc(*x*) = sin(π*x*)/π*x*, and *x*_1_ and *x*_2_ are the coordinates at the image plane. The beam size and coherence length in the above equation are the values at the final image plane. They can be derived from source parameters according to equation (13).

Before we perform the detailed calculation using the above convolution relation, let us consider some extreme cases qualitatively. When the aperture size *L* → +∞, the second term of the convolution operation in equation (16) is δ(*x*_1_/λ*z*)δ(*x*_2_/λ*z*), where δ(*x*) is the Dirac delta function. Using the properties of the Dirac delta function, one can confirm that the CSD function at the image plane in this case is the ideal one, *G*_*z*_(*x*_1_, *x*_2_) = *G*_0*z*_(*x*_1_, *x*_2_). Next, let us consider the nano-focusing scheme. Table 1 ▸ shows that the demagnification factor is about 1184, which leads to an ideal focal spot size of about 7.76 nm. However, the second term of equation (16) will be larger than this small ideal spot size. The limitation from the aperture could be considered as follows. The phase term exp[−*ik*(*x*_1_^2^ − *x*_2_^2^)/2*z*] in equation (16) has negligible impact and can be ignored safely for the nano-focusing case. Supposing ξ_*x*_ 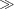 σ_*x*_, within the range of significant beam intensity, the coherence term exp[−(*x*_1_ − *x*_2_)^2^/2ξ_*x*_^2^] is approximately 1. Then the intensity profile at the final image plane is the convolution of the ideal intensity with the square of the sinc function. Due to the limited optic aperture, the final spot size is larger than the ideal value. This is the so-called diffraction-limited case.

**Figure 2:**
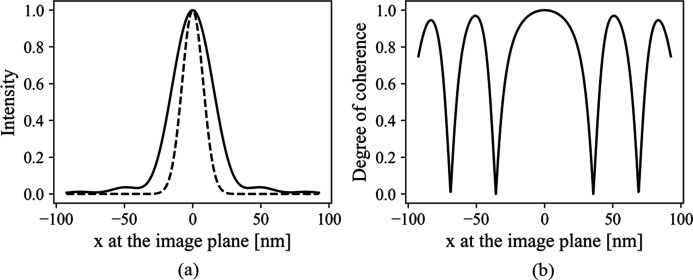
(*a*) The normalized intensity of perfect optics (dashed line) and after a finite aperture of 0.4 mm (solid line). (*b*) The spectral degree of coherence function after the finite aperture. One of the coordinates in SDC function is set to be at the centre.

Fig. 2 ▸ gives a typical nano-scale intensity profile and the SDC function modulated by an aperture size of 0.4 mm. The results are obtained from equation (16) for the nano-focusing mode listed in Table 1 ▸. As shown in Fig. 2 ▸(*a*), the final intensity profile is broadened by the finite aperture. Moreover, Fig. 2 ▸(*b*) shows that, within the area of sufficient intensity at the final image plane, the wave is highly coherent.

**Figure 3:**
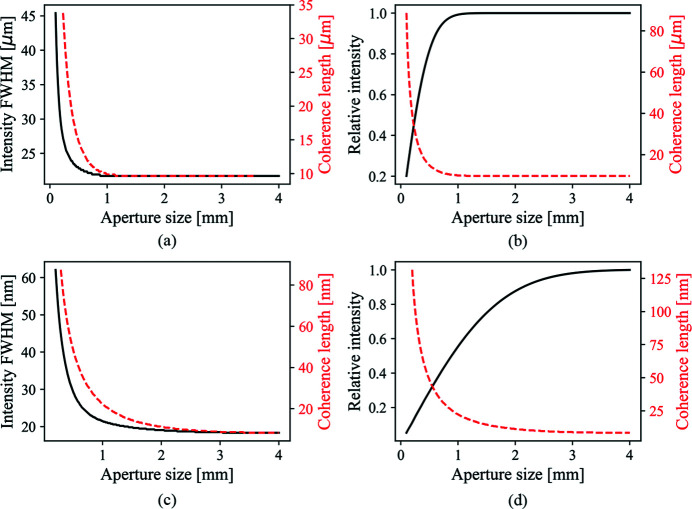
The FWHM of intensity profile and coherence length as a function of aperture size for 1:1 focusing (*a*) and nano-focusing (*c*). The loss of intensity as a drawback of increasing coherence length by decreasing the aperture size are shown for the 1:1 focusing case (*b*) and nano-focusing case (*d*), respectively.

Singer & Vartanyants (2014 ▸) have discussed the influence of focusing optics of varying aperture sizes on the focal spot size and coherence properties if the aperture is Gaussian in shape. Here, we also calculated the effects of different rectangular aperture sizes with little difficulty using equation (16). However, equation (16) can deal with apertures of any shape. As long as an appropriate complex transfer function for the aperture can be defined, the impact on the coherence properties can be calculated using equation (8). We use the full width at half-maximum (FWHM) value for the central peak of the SDC function as the coherence length. In addition, we use the FWHM of the intensity profile as the beam size at the image plane. Two focusing schemes listed in Table 1 ▸ are considered. Figs. 3 ▸(*a*) and 3(*c*) show that the larger the aperture size, the shorter the coherence length. The limiting case is the coherence length obeying the equation (13). On the other hand, a larger aperture size will result in a smaller focal spot size. The limit here is the ideal focus size according to equation (13). One must also consider the sacrifice of beam intensity when decreasing the aperture size in order to obtain higher coherence. Figs. 3 ▸(*b*) and 3(*d*) show the relation between the beam intensity and the beam coherence. At one limit is large coherence length with small intensity. At the other limit is the ideal optic with no loss of intensity and the coherence length given by equation (13).

Higher focal intensity and larger coherence length are always mutually exclusive, as are smaller focus size and larger coherence length. Using the theoretical approach proposed in Section 2, we can assess the trade-off between these considerations relatively easily as shown in Fig. 3 ▸.

### 3.3. Focusing mirror with height error distribution   

The X-ray mirror is one of the most widely used optical elements for focusing. Apart from the aperture size, surface height error is another common source of focal spot degradation. It is well known that the height errors across the non-ideal optical surface *h*(*x*_s_) with the mirror surface coordinate *x*_s_ give rise to a phase shift ΔΦ_h_. If the radiation wavelength is λ and the grazing incident angle is θ, the phase shift could be written as follows,
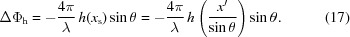
In equation (17), the optical surface coordinate *x*_s_ and the optical surface exit plane coordinate *x*′ (see Fig. 1 ▸ for exit plane) has the relation of *x*′ = *x*_s_sinθ. The complex transfer function at the exit plane is
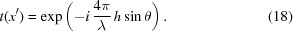

*h* in the above equation could be regarded as a function of the mirror surface coordinate *x*_s_ or the exit plane coordinate *x*′. Once we have equation (18) for the complex transfer function, we could use the theory outlined in Section 2 again for the evaluation of coherence properties degraded by the mirror surface height error.

**Figure 4:**
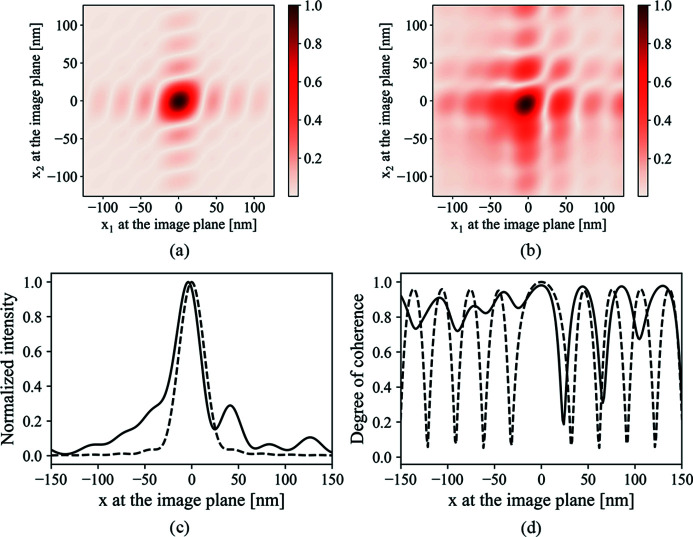
(*a*) The normalized CSD function from an ideal surface. (*b*) The normalized CSD function from a surface with figure error. The figure error used is shown in Fig. 5 ▸(*a*). (*c*) The ideal (dashed line) and distorted (solid line) normalized intensity profile. The ideal intensity profile already accounts for the finite aperture size. (*d*) The ideal (dashed line) and distorted (solid line) SDC function.

**Figure 5:**
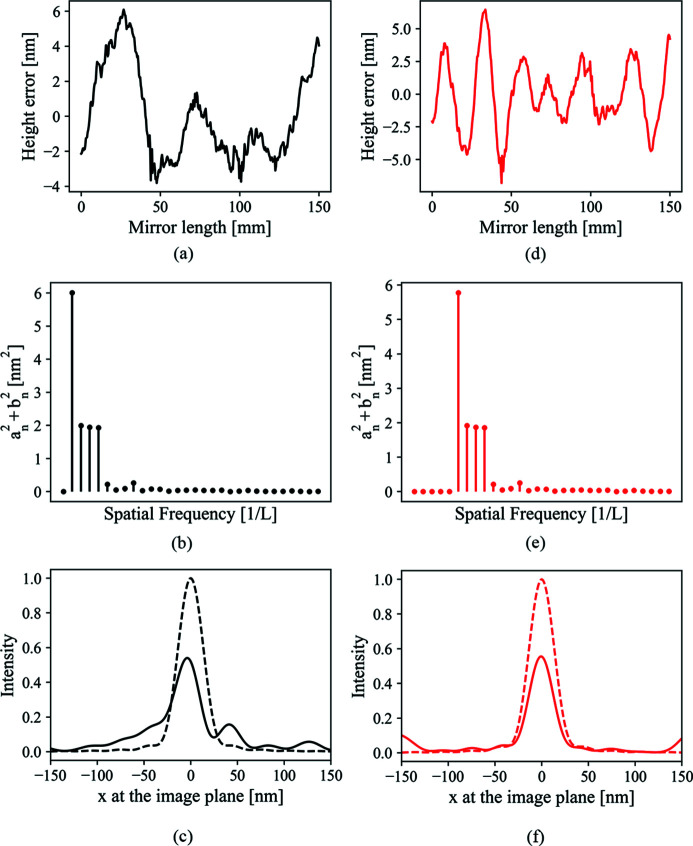
(*a*, *d*) Two types of figure height error distributions. (*b*, *e*) The corresponding square sum of Fourier components |*a*_*n*_|^2^ + |*b*_*n*_|^2^. Only the first 30 terms are shown here. (*c*,*f*) The intensity profiles resulting from these two errors. The dashed line is the ideal intensity profile.

Fig. 4 ▸ shows the normalized CSD function, the intensity profile and the degree of coherence with and without degradation by a theoretical mirror surface height error. The nano-focusing scheme described in Table 1 ▸ is used. The mirror length is 150 mm with a grazing angle of 3 mrad. The aperture size effect has been considered for the ideal surface height distribution. The surface height error for this calculation is shown in Fig. 5 ▸(*a*). The rest of Fig. 5 ▸ will be discussed later to show a better definition of the spatial frequencies at which height errors degrade the focus most severely. One obvious impact shown in Fig. 4 ▸(*c*) is the severely distorted intensity profile. We point out that this is mainly due to the low spatial frequency components of the mirror surface error.

In the remainder of this section, we will give a physical explanation for the intensity profile distortion following the theoretical outline described in Section 2. The mirror height error function *h*(*x*′) with respect to the exit plane coordinate can be decomposed into its Fourier series,
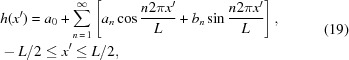
with
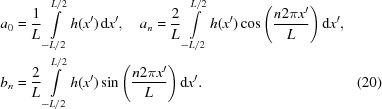
Using the convolution theorem of the Fourier transform, the Fourier transform term in equation (10) can be written as a successive convolution of Fourier transforms of the phase term caused by cosine and sine functions. This prompts us to treat the simple sinusoidal and cosinusoidal height error distribution first.

Assume the mirror surface error has a cosinusoidal distribution
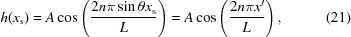
where *A* is the half of peak-to-valley (P–V) value of the surface height error, *x*_s_ and *x*′ are the coordinates on the mirror surface and the exit plane, respectively, the mirror length is *L*/sinθ, and *L* is the projected mirror length on the exit plane. The period of the surface error distribution projected on the exit plane is *L*/*n*. The asymptotic expansion of the intensity profile affected by the cosinusoidal distribution is
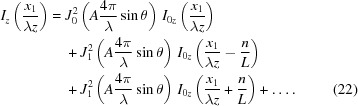

**Figure 6:**
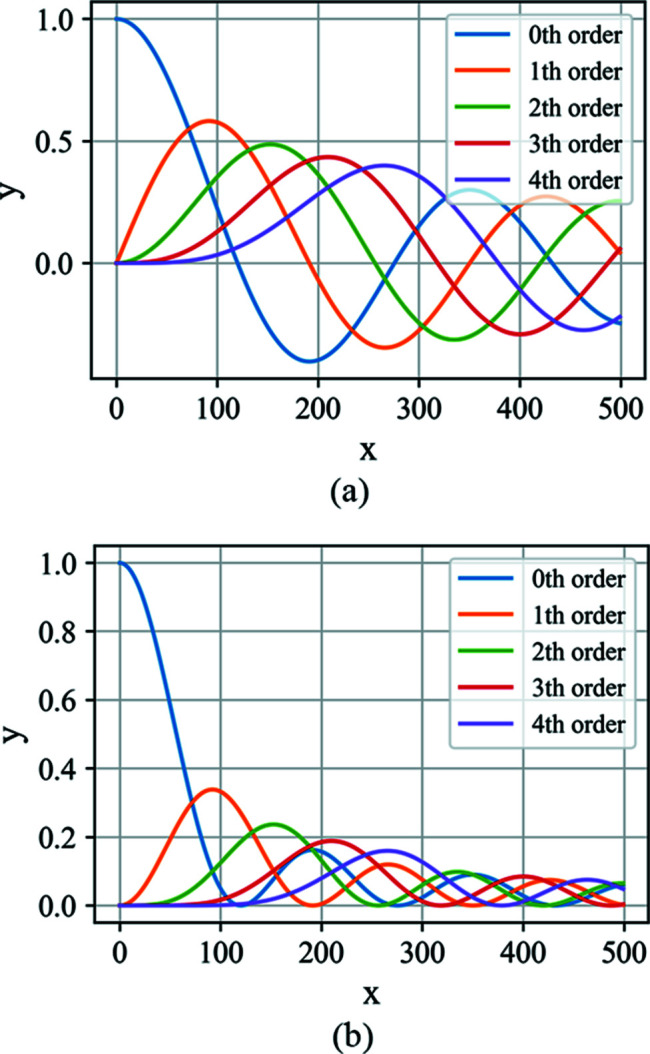
(*a*) The Bessel functions of the zeroth to fourth order. (*b*) The square of the Bessel functions.

*J*_*i*_(*x*) in the above equation are Bessel functions of the first kind. Fig. 6 ▸ shows these Bessel functions and their squared values. Under the ultra-smooth mirror surface assumption, *A* ≤ λ/4πsinθ, the higher-order Bessel functions are much smaller than the lower-order Bessel functions (see Appendix *B* [App appb] for the properties of Bessel functions). Equation (22) tells us that a cosinusoidal height error will replicate the damped ideal peak at positions determined by the spatial frequency *n*/*L*. The intensity of the damped central peak and the nearest side peaks are determined by zeroth- and first-order Bessel functions, respectively. The higher orders of the above expansion correspond to higher orders of Bessel functions. A similar result has been given by Raimondi & Spiga (2015 ▸).

Similarly, the intensity profile degradation due to the sinusoidal distribution,
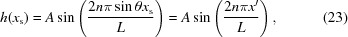
can be expressed as
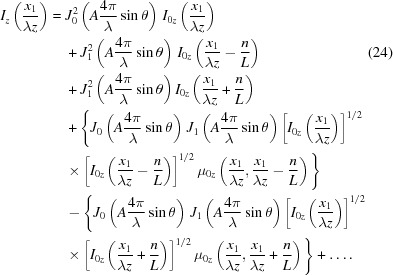
For the derivation of equations (22) and (24), see Appendix *B* [App appb].

Two obvious properties can be spotted from the intensity profile of the two simple models. One is that the central intensity drops according to the P–V values of the height error distribution. This can be seen from the first term of the asymptotic expansion of equations (22) and (24). The central intensity is scattered according to the square of the Bessel function. The scattered intensity is approximately
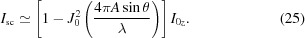
Another property is that the side peaks at the image plane appear at the position
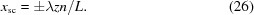
The complex transfer function for a real surface combines equations (18) and (19). The real surface impact can be determined by successive convolution with single spatial frequency terms from the Fourier transform of the real surface. When one uses the asymptotic expansion for each individual single spatial frequency term as discussed above, one finds the asymptotic expansion of the real surface height error by the successive application of equations (22) and (24) for every component of its Fourier transform. The ideal intensity distribution for a certain Fourier transform component is the distorted one by its previous component. Thus, the central peak intensity from the imperfect surface decreases as
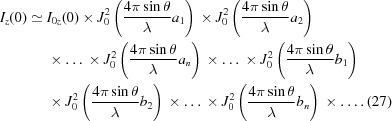
In the above equation, *a*_1_, *a*_2_, *a*_*n*_,…, *b*_1_, *b*_2_, *b*_*n*_,… are the coefficients of the Fourier transform of the mirror surface error function. For the ultra-smooth mirror used for X-rays, *a*_*i*_, *b*_*i*_ in the above equation are quite small: |*a*_*i*_|, |*b*_*i*_| ≤ λ/4πsinθ. The above equation then leads to the well known result (Als-Nielsen & McMorrow, 2011 ▸; Harvey, 1995 ▸)
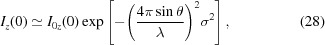
where σ is the RMS value of the height error for the spatial frequency range within which the scattered intensity falls outside the central cone (Shi *et al.*, 2016 ▸). For a detailed derivation of equation (28) from equation (27), see Appendix *C* [App appc].

It is well known that the low spatial frequencies give rise to the side peaks, which distort the focal spot shape. The commonly used definition is the critical length of the mirror, as discussed by various researchers (Church & Takacs, 1993 ▸; Pardini *et al.*, 2015 ▸; Shi *et al.*, 2016 ▸),
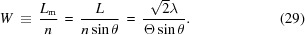
In the above equation, *L*_m_ is the mirror length and Θ is defined as the angular size of the image. The previous researchers proposed that when the spatial frequency is larger than that defined by 1/*W*, the scattered intensity will have limited impact on the focus shape. However, the exact value of Θ is slightly ambiguous. We point out here that this definition is merely another form of equation (26), with the note that Θ has the same unit of radian as *x*_s_/*z* in equation (26). Usually Θ is set as *S*/*z*, where *S* is the image size. We show this value will not guarantee us an undistorted intensity profile. Comparing equation (29) with equation (26), if Θ = *S*/*z*, the spatial frequency of 1/*W* will contribute a side peak at position *x*_sc_ = 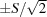. This is not far enough from the central cone, and it will distort the focus shape. We propose to use the equivalent equation (26) to determine the lowest spatial frequency to ensure the side peaks fall far enough from the central focal spot. The coordinate *x*_sc_ in the image plane in equation (26) can be scanned over several times the full image size to secure a safe spatial frequency range.

Fig. 5 ▸ shows the focus degradation due to different spatial frequency ranges. Two mirror surface height error distributions are shown, one in Fig. 5 ▸(*a*) and the other in Fig. 5 ▸(*d*). Their power spectral density (PSD) functions (Alcock *et al.*, 2010 ▸), shown in Figs. 5 ▸(*b*) and 5(*e*), respectively, differ only in an overall shift of four spatial frequency units. The lowest spatial frequency in Fig. 5 ▸(*d*) will form the side peaks around the position of ±151.6 nm at the image plane according to equation (26). Fig. 5 ▸(*f*) shows that this range of spatial frequency indeed has little impact on the intensity profile except a decrease of the central intensity. Fig. 5 ▸(*c*) shows that the low frequency, which falls within the central intensity profile according to equation (26), plays the dominant role in focus shape distortion.

**Figure 7:**
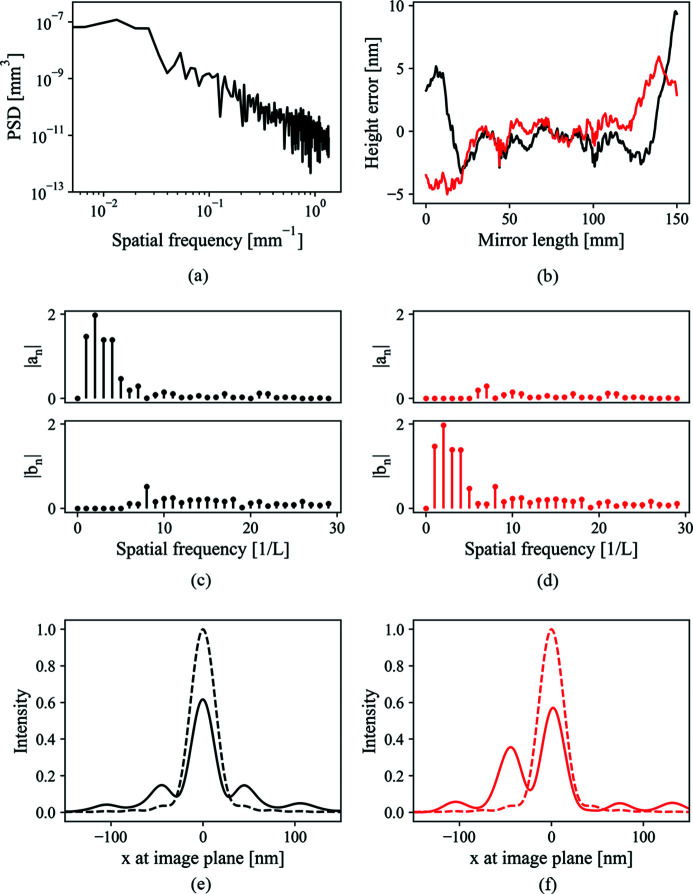
(*a*) PSD function of the distorted mirror surface. (*b*) Two surface height errors with same PSD function. (*c*, *d*) Ratio of sinusoidal and cosinusoidal terms in their Fourier decomposition for the corresponding two errors. (*e*, *f*) Intensity profiles distorted due to the two types of errors. (*e*) Intensity after a mirror with a height error consisting of mainly cosinusoidal terms in the low frequency range. (*f*) Intensity after a mirror with a height error consisting of mainly sinusoidal terms in the low frequency range.

On the other hand, the focal spot shape will be severely distorted by the low frequency range. Even with a simple sinusoidal or cosinusoidal surface distribution, which have only a single spatial frequency, equations (22) and (24) show different redistributions of the scattered intensity. This fact prompts us to consider that, for highly coherent beam such as DLSR or FEL, even the full knowledge of the PSD function does not tell the whole story. In the following, the mirror surface height error distributions have the same PSD function as shown in Fig. 7 ▸(*a*).

Fig. 7 ▸(*b*) gives two figure errors with the same PSD function. The only difference is the ratio of sinusoidal and cosinusoidal terms in their Fourier decomposition. This is shown in Figs. 7 ▸(*c*) and 7(*d*). The performance of these two distorted surfaces are shown in Figs. 7 ▸(*e*) and 7(*f*). Fig. 7 ▸(*e*) shows that the mirror surface error mainly decreases the central intensity while preserving the intensity profile. In Fig. 7 ▸(*f*), on the other hand, the main peak is split into two nearly equal parts. This example shows that even full knowledge of the PSD function cannot guarantee us an accurate judgement of the mirror quality. This is due to the different behaviour of sinusoidal and cosinusoidal terms according to equations (22) and (24).

The above physical analysis of the mechanism for intensity profile distortion shows that the distortion mainly comes from the low spatial frequency range. This frequency range relates to the specific optical layout and can be calculated using an analytical expression. Within the low spatial frequency range, the distortion of the intensity profile is complex, and the PSD function alone could not predict its impact. This indicates that, for a DLSR or FEL beamline, each mirror should be treated as a special case, especially in the low frequency range. The theoretical approach proposed in Section 2 provides a tool for beamline designers to quickly evaluate the impacts of imperfect mirrors when metrology data are available. Besides, the fact that the low spatial frequency plays the killer role in focus shape distortion makes adaptive optics such as bimorph mirrors (Alcock *et al.*, 2019*a* ▸ *b* ▸, 2013 ▸; Sutter *et al.*, 2019 ▸) or refractive corrector (Laundy *et al.*, 2019 ▸) an ideal solution for the focus shape correction.

## 4. Summary   

In this paper, we have developed a tool to rapidly evaluate the performance degradation due to the imperfection of an optical element. The effects due to the finite optical aperture size and mirror surface height error distribution have been discussed. These two cases are used to demonstrate the applicability of the presented theory. Moreover, by applying the proposed theory, we have also given a physical explanation of the intensity profile distortion caused by the mirror surface height error distribution. A better definition of low frequency range has been proposed. Instead of using image size, we proposed to use the coordinate on the image plane as the parameter for low frequency range determination. Within the low frequency range, one example is given to demonstrate that, for highly coherent sources, even the full knowledge of the PSD function cannot guarantee an accurate judgement of the mirror quality. The proposed theoretical approach could help beamline designers to evaluate optical element errors before performing more detailed, but also more computationally demanding, wave optics simulations. It is also useful for when a large amount of metrology data need to be screened.

Although we use the GSM in this paper, the application of the presented approach is not limited to this model. As long as we obtain the CSD function after the ideal optic, the proposed approach can be applied. As a result, the presented theoretical approach in this paper has a wide range of applications.

## Figures and Tables

**Table:** 

Source parameters			
Photon energy	10 keV	Source divergence (H)	4.97 µrad
Source size (H)	9.14 µm	Coherence length (H)	4.07 µm

**Table:** 

	Diffraction-limited focusing	1:1 micro-focusing
Source distance	130.25 m	38.5 m
Image distance	0.11 m	38.5 m
Demagnification factor	1184	1

## Appendix A Propagation of cross spectral density function through non-ideal surface

In this section we consider only the one-dimensional cross spectral density function. For an ideal surface, the propagation of the cross spectral density is described by

*G*_0_(*x*_1_, *x*_2_) is the CSD function on the exit plane right after the ideal optical surface, while *G*_0*z*_(*x*_1_, *x*_2_) is the ideal CSD function at the image plane. To treat a non-ideal surface, one must include its transfer function in the propagated CSD function as follows,

*G*_*z*_(*x*_1_, *x*_2_) is the non-ideal CSD function at the image plane, *t*(*x*′) is the complex transfer function for a non-ideal surface and *t**(*x*′) is its conjugate. In the following, we try to establish a relationship between *G*_0*z*_(*x*_1_, *x*_2_) from equation (30) and *G*_*z*_(*x*_1_, *x*_2_) from equation (31). First, we state the usual Fourier transform*F_υ_* and the inverse Fourier transform ,

Besides, ‘*’ denotes the usual convolution operation,

Using the definition of the Fourier transform, equation (30) can be expanded as

In equation (34),  means performing the Fourier transform from  into *x*_1_/λ*z*,  means performing the inverse Fourier transform from  into *x*_2_/λ*z*. Using a similar approach, we expand equation (31) as

Applying the convolution theorem of the Fourier transform (Goodman, 2005 ▸), equation (35) can be further written as

The convolution in equation (36) is over *x*_1_. To simplify the discussion, we denote *K*(*x*_1_, ) and *H*(*x*_1_, ) as

Thus, equation (36) can be stated as

where *G*(*x*_1_, ) and *H*(*x*_1_, ) are defined in equation (37). Using Fubini’s theorem^1^ (Rudin, 1987 ▸), the order of the last integration in equation (38) can be switched, giving the following equation,

In equation (39), we use the convolution theorem of the Fourier transform again, this time convoluting over *x*_2_. Let *L*(ξ, *x*_2_) and *M*(*x*_1_ − ξ, *x*_2_) be defined as

Using the definition of equation (40), equation (39) is rewritten as

In equation (41), the symbol ⊗ denotes the 2D convolution. According to the definition of *L*(*x*_1_, *x*_2_) in equation (40) and the definition of *G*(*x*_1_, *x*_2_) in equation (37), we explicitly rewrite *L*(*x*_1_, *x*_2_) as

Furthermore, *M*(*x*_1_ *, x*_2_) is written as

With the help of the above equations, we rewrite equation (41) as

Comparing equation (44) with equation (34), we establish a relationship between the CSD function *G*_*z*_(*x*_1_, *x*_2_) for the non-ideal surface and the CSD function *G*_0*z*_(*x*_1_, *x*_2_) for the ideal surface by a 2D convolution. Explicitly, it is shown that

## Appendix B Intensity profile degradation from cosinusoidal and sinusoidal figure error

In order to determine the effect of cosinusoidal and sinusoidal figure errors on intensity profiles, we need some useful properties of Bessel functions of the first kind. The following identities are given for reference. Detailed information can be found in the literature (Wang & Guo, 1989 ▸; Whittaker & Watson, 1996 ▸).

The relationship between the positive integer order *J*_*n*_(*x*) and its negative counterpart is

The sum of Bessel functions has the following properties,

Sums over *J*_*n*_(*x*) are also related to trigonometric functions,

Equation (48) is used for the derivation of the Fourier transform of cosinusoidal and sinusoidal mirror surface height error functions.

The cosine height error function affects the intensity profile at the image plane according to the convolution operation in equation (10). The Fourier transform and inverse transform terms are

As in previous sections,  denotes a Fourier transform from  into *x*_1_/λ*z*. Similarly,  denotes an inverse Fourier transform from  into *x*_2_/λ*z*. By combining the above equation and equation (10), the intensity distribution at the image plane when the mirror has a cosinusoidal surface height error can be written as

In order to preserve the wavefront as much as possible, the mirrors required for X-ray optics are usually ultra-smooth. That means the P–V value of 2 A should be quite small. Fig. 6 ▸ shows the figure of Bessel functions and their squared values. Under the ultra-smooth mirror surface assumption, the high-order Bessel functions are much smaller than the low-order Bessel functions. This leads to the asymptotic expansion of equation (22),

Similarly, for a sinusoidal figure error distribution, the Fourier transform and inverse transform terms for the sine function in equation (10) are

The intensity distribution at the image plane when the mirror has a sinusoidal surface height error can be written as

The first few terms of the asymptotic expansion of (24) are

## Appendix C From Bessel function to exponential function

The squared zeroth-order Bessel function  can be approximated by the Gaussian function exp(−*z* ^2^/2). The power series of  is (Wang & Guo, 1989 ▸; Whittaker & Watson, 1996 ▸)

Around *z* = 0, the above equation can be approximated by

By comparing with the series expansion of the Gaussian function exp(−*z* ^2^/2), the squared zeroth-order Bessel function at around *z* = 0 can be approximated as

For the ultra-smooth mirrors used for X-rays, *a*_*i*_, *b*_*i*_ in equation (27) are quite small, |*a*_*i*_|, |*b*_*i*_|  λ/4πsinθ. Then equation (27) can be approximated as

From Parseval’s equality (Boggess & Narcowich, 2015 ▸), the above result is equivalent to the well known one (Als-Nielsen & McMorrow, 2011 ▸; Harvey, 1995 ▸),

where σ is the RMS value of the mirror height error.
